# Analysis of Publicly Funded Reinsurance—Government Spending and Insurer Risk Exposure

**DOI:** 10.1001/jamahealthforum.2021.1992

**Published:** 2021-08-13

**Authors:** Maria Polyakova, Vinayak Bhatia, M. Kate Bundorf

**Affiliations:** 1 School of Medicine, Stanford University, Stanford, California; 2 National Bureau of Economic Research, Cambridge, Massachusetts; 3 Carnegie Mellon University, Pittsburgh, Pennsylvania; 4 Sanford School of Public Policy, Duke University, Durham, North Carolina

## Abstract

This analysis compares the design of section 1332 reinsurance policies across states based on their potential for reducing insurer risk exposure and likely level of government spending

## Introduction

States have received approval from the Centers for Medicare & Medicaid Services to operate publicly financed reinsurance programs through section 1332 state innovation waivers.^1^ Reinsurance—insurance for insurers—protects insurers from the risk of unusually high enrollee spending.^2^ Publicly financed risk protection for insurers can both promote insurer participation in markets and reduce their incentives to avoid high-risk enrollees.^3^ In this study, we compare the design of section 1332 reinsurance policies across states based on their potential for reducing insurer risk exposure and likely level of government spending.

## Methods

We used an algorithm (eAppendix in the Supplement) to simulate 12 operational state-based reinsurance programs, 3 federal reinsurance policies adopted as part of the Affordable Care Act, and 2 benchmark commercial stop-loss policies for a standardized population (Table^1^).^4^ We examined 2 metrics for each program: (1) total government spending on reinsurance claims and average insurer liability (the sum of government spending and insurer liability is a constant), and (2) insurer risk exposure, measured by the coefficient of variation—the standard deviation divided by the mean of per-enrollee insurer liability. Because this study did not meet the definition of human subjects research as defined by the Common Rule (45 CFR §46), it was not reviewed by an institutional review board.

**Table.** Parameters and Timeline of the Simulated Reinsurance Programs^a^

Reinsurance program	Reinsurance parameters	Approval date	Implementation year
Federal reinsurance policy			
2014	100% Of claims $45 000-$250 000	NA	2014
2015	55.1% Of claims $45 000-$250 000	NA	2015
2016	52.9% Of claims $90 000-$250 000	NA	2016
Alaska	100% Of paid claims of individuals with 1 of 33 conditions	7/7/17	2018
Colorado	Either 45%, 50%, or 85% of claims $30 000-$400 000, depending on the rating area	7/31/19	2020
Delaware	75% Of claims $65 000-$215 000	8/20/19	2020
Maine	90% Of claims between $47 000-$77 000 and 100% afterward; insurers must cede policies	7/30/18	2019
Maryland	80% Of claims $20 000-$250 000	8/22/18	2019
Minnesota	80% Of claims $50 000-$250 000	9/22/17	2018
Montana	60% Of claims $40 000-$101 750	8/16/19	2020
New Jersey	60% Of claims $40 000-$215 000	8/16/18	2019
North Dakota	75% Of claims $100 000-$1 000 000	7/31/19	2020
Oregon	50% Of claims $95 000-$1 000 000	10/19/17	2018
Rhode Island	50% Of claims $40 000-$97 000	8/26/19	2020
Wisconsin	50% Of claims $50 000-$250 000	7/29/18	2019
Commercial individual stop-loss	100% Of claims ≥$250 000	NA	NA

Abbreviation: NA, not applicable.

^a^ The Table records reinsurance programs that were simulated in the article. Reinsurance coverage parameters, approval dates, and implementation dates are listed elsewhere.^1^

## Results

Each program was associated with greater government spending, lower insurer liability, and greater insurer risk protection compared with no reinsurance (Figure). Large differences existed, however, across programs. State and federal reinsurance policies were far from the frontier, defined as a policy that achieves the maximum simulated risk protection for insurers for a given level of public funding. The frontier policy is full insurance above an attachment point.

**Figure.**
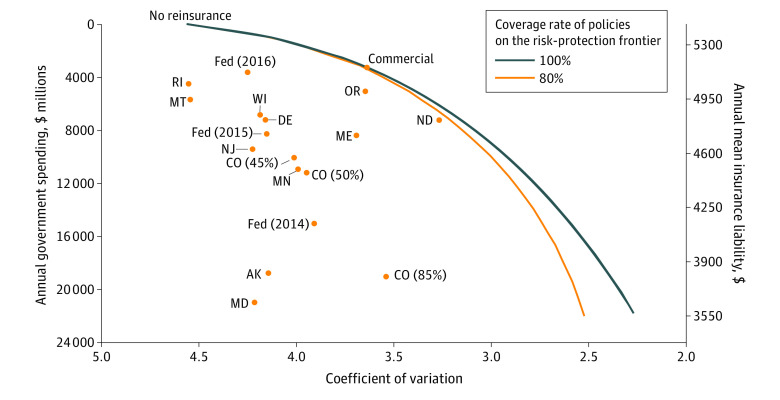
Trade-off Between Risk Protection for Insurers and Public Spending The Figure plots annual government spending against coefficient of variation in insurer liability for each of the simulated reinsurance policies. The lines trace the risk protection that the government could provide for each level of annual public spending if public reinsurance contracts followed the commercial structure with either 80% (dark blue) or 100% (orange) coinsurance rates. Axes are reversed to represent the trade-offs that policy makers face. The association between the mean insurer liability and government spending is mechanical in the simulation because the sum of the 2 is fixed in each simulation. As the government spends more, insurer liability falls. Fed indicates federal reinsurance policy; the Fed and states’ positions on the graph and the Colorado (CO) percentages are detailed in the Table.

While program parameters differed across states, they resulted in remarkably similar—and relatively low—simulated insurer risk protection (Figure). The coefficient of variation fell below 4.0 for only 4 states. In most states, insurer risk protection could have been greater for the same level of government spending. For example, Delaware and North Dakota’s programs were associated with similar levels of government spending, yet North Dakota’s was associated with greater insurer risk protection. Conversely, Rhode Island and Maryland’s programs were associated with similar insurer risk protection, yet Maryland’s was associated with substantially higher government spending.

## Discussion

Results of this analysis showed that states could modify their reinsurance programs to maintain their level of spending and increase insurer risk protection, strengthening incentives for insurer entry and reducing incentives to avoid high risks. However, greater insurer risk protection has possible downsides. States may not be better positioned to bear catastrophic risk than private reinsurers and may be reluctant to crowd out private reinsurance, a market that has recently been revived by the elimination of the lifetime cap on covered expenditures. More comprehensive reinsurance could also increase spending by reducing incentives for insurers to manage care for the sickest patients.

Public spending on reinsurance can also indirectly subsidize insurance for consumers. Indeed, lowering premiums, particularly for higher-income exchange enrollees who do not receive income-based subsidies, was one of the explicit objectives of reinsurance programs. However, the American Rescue Plan has extended direct premium subsidies to more families, reducing the size of this population by more than 50%.^5^ The relative costs and benefits of spending public dollars through direct premium subsidies and publicly funded reinsurance are an open question. A general concern in both cases is whether government-financed payments ultimately reach consumers. Limited insurer competition may lead to limited pass-through of any subsidies to consumers.^6^

A limitation of this study is that it does not provide empirical evidence on the effects of reinsurance programs. Instead, we provide insights into the trade-offs facing policy makers when considering whether and how to provide publicly financed reinsurance.

## Notes

Supplement. eAppendix. Supplemental materials.
