# Supplementary material for: Analysis of Publicly Funded Reinsurance—Government Spending and Insurer Risk Exposure
Source: JAMA Health Forum. 2021 Aug 13;2(8):e211992. doi: 10.1001/jamahealthforum.2021.1992 (PMC8796983; doi:10.1001/jamahealthforum.2021.1992)
Supplement: Supplement. — eAppendix. Supplemental materials. [file jamahealthforum-e211992-s001.pdf]

Supplemental Online Content

Polyakova M, Bhatia V, Bundorf MK. Analysis of publicly funded reinsurance—government spending and insurer risk exposure. *JAMA Health Forum*. 2021;2(8):e211992. doi:10.1001/jamahealthforum.2021.1992

eAppendix. Supplemental materials

This supplemental material has been provided by the authors to give readers additional information about their work.

© 2021 Polyakova M et al. *JAMA Health Forum.*

**Analysis of Publicly Funded Reinsurance:
Government Spending and Insurer Risk Exposure**

Maria Polyakova, PhD[1]

Vinayak Bhatia, BA[2]

M. Kate Bundorf, MBA, MPH, PhD[3]

SUPPLEMENTAL MATERIALS

Standardized population (follows discussion in Polyakova, Hua, Bundorf, 2017)

As we outline in Polyakova, Hua, Bundorf (2017), our simulation model uses 1 percent random sample from the Truven MarketScan database, restricting our analysis to the 173,069 people ages 18–64 with information for both 2012 and 2013—just before the launch of the ACA Marketplaces. The data provide individual-level, two-year histories of health care use, including inpatient and outpatient medical procedures and services, as well as prescription pharmaceuticals. Our main goal is to compute how each of 2018 Marketplace plans would have covered these utilization histories. We apply 2018 federally-facilitated Health Insurance Marketplace plan designs to 173,069 health profiles to estimate the out-of-pocket expenditures these individuals would have incurred had they been enrolled in these plans and chose to utilize exactly the same medical services.

Computation of insurer and consumer liability (follows discussion in Polyakova, Hua, Bundorf, 2017)

Our data for plan information came from the Centers for Medicare and Medicaid Services—specifically, the following 2018 Health Insurance Marketplace Public Use Files: the Benefits and Cost Sharing, Rate, Plan Attributes, Service Area, and Machine-readable URL Public Use Files. Collectively, these files provide rich information about each plan's coverage of various types of visits and procedures, premiums, and region of coverage and its detailed formulary. We considered only medical plans that were in the Marketplace and were not in the Small Business Health Options Program.

We took into account many details of plan benefit design in our calculations. First, we assumed that an enrollee paid the full cost of health care services out of pocket until he or she met the plan-specific deductible, with the exception of preventive services as outline below.

[1] Maria Polyakova is the corresponding author and an Assistant Professor of Medicine in the Center for Health Policy, Stanford University and a faculty research fellow at the National Bureau of Economic Research.
[2] Vinayak Bhatia is a doctoral candidate at Carnegie Mellon University.
[3] M. Kate Bundorf is the J. Alexander McMahon Distinguished Professor of Health Policy and Management at Duke University and a research associate at the National Bureau of Economic Research

© 2021 Polyakova M et al. *JAMA Health Forum.*

Second, for spending that exceeded the deductible, we allowed patient cost-sharing payments to depend on the benefit category of the claim and the corresponding copay or coinsurance for the service rendered. These amounts vary within and across plans depending on the type of medical procedure or service. Using a combination of MarketScan data variables, we classified the claims for each enrollee into more than a hundred benefit categories. We then applied the cost sharing associated with the benefit category to the claim's allowed amount. When the allowed amount for a claim was less than the copay amount for the service, we assumed that the enrollee paid the entire claim amount out of pocket. To identify ACA-mandated preventive care or screening services that are exempt from cost sharing, we used EmblemHealth, CIGNA, and BCBS guidelines to flag claims based on patient age, sex, frequency of service, and specific procedure codes. These guidelines are aligned with recommendations from the US Preventive Services Task Force, the Health Resources and Services Administration, and the Advisory Committee on Immunization Practices.

Finally, once a patient's out-of-pocket spending reached the plan's maximum, we assumed that the patient no longer made out-of-pocket payments for services, unless the service was a noncovered procedure.

Computation of actuarially fair premiums

We defined the actuarially fair premium to equal the average insurer liability in the standardized population. We computed the average insurer liability for each of 1,924 plans offered on 2018 federally-facilitated Marketplaces for which formulary and benefit information was available. We then took an unweighted average of this liability estimate across all 1,924 plans to arrive at the aggregate actuarially fair premium that is reported in Figure 1 of the manuscript.

Computation of government spending

We applied reinsurance rules as proposed by each state to each Marketplace plan in our simulation to compute plan-level government spending for the standardized population. We then took an unweighted average of government liability across all plans. We next rescaled the average government liability within our standardized population by enrollment counts in the ACA federally-facilitated Marketplaces.

Computation of insurer risk measure

We defined insurer risk as the coefficient of variation (CV) of the claims that insurers face. For each plan in our simulation we computed plan-specific CV by dividing the standard deviation in plan-specific insurer liability in our standardized population by the mean of plan-specific insurer liability. We then took an unweighted average of plan-specific CVs to arrive at one insurer risk measure that is reported in Figure 1 of the manuscript. CV of zero implies that there is no variation in insurer cost across individuals, so the insurer faces no risk. A higher CV implies increasingly more risk.

© 2021 Polyakova M et al. *JAMA Health Forum.*
